# A Quantum-like Approach to Semantic Text Classification

**DOI:** 10.3390/e27070767

**Published:** 2025-07-19

**Authors:** Anastasia S. Gruzdeva, Rodion N. Iurev, Igor A. Bessmertny, Andrei Y. Khrennikov, Alexander P. Alodjants

**Affiliations:** 1 National Center for Cognitive Research, National Research University for Information Technology, Mechanics and Optics (ITMO), St. Petersburg 197101, Russia; prog.anastasia@gmail.com (A.S.G.); alexander_ap@list.ru (A.P.A.); 2 Faculty of Software Engineering and Computer Systems, National Research University for Information Technology, Mechanics and Optics (ITMO), St. Petersburg 197101, Russia; 266312@edu.itmo.ru (R.N.I.); bessmertny@itmo.ru (I.A.B.); 3 International Center for Mathematical Modeling in Physics, Engineering, Economics and Cognitive Science, Linnaeus University, S-35195 Vaxjo-Kalmar, Sweden

**Keywords:** quantum-like heuristic algorithms, text classification, sentiment analysis, interference, vector-space language model

## Abstract

In this work, we conduct a sentiment analysis of English-language reviews using a quantum-like (wave-based) model of text representation. This model is explored as an alternative to machine learning (ML) techniques for text classification and analysis tasks. Special attention is given to the problem of segmenting text into semantic units, and we illustrate how the choice of segmentation algorithm is influenced by the structure of the language. We investigate the impact of quantum-like semantic interference on classification accuracy and compare the results with those obtained using classical probabilistic methods. Our findings show that accounting for interference effects improves accuracy by approximately 15%. We also explore methods for reducing the computational cost of algorithms based on the wave model of text representation. The results demonstrate that the quantum-like model can serve as a viable alternative or complement to traditional ML approaches. The model achieves classification precision and recall scores of around 0.8. Furthermore, the classification algorithm is readily amenable to optimization: the proposed procedure reduces the estimated computational complexity from $O(n^{2})$ to O(n).

## 1. Introduction

Text analysis is an important element of evaluating distributed intelligent systems (DISs), which combine natural and artificial intelligence agents [1,2,3]. Short messages, which agents can modify during their communication, are not only carriers of some contextual information but also a way to convey emotions and moods in DIS. Consequently, the effective functioning of DIS, which also includes elements of decision making, performed under uncertainty, should necessarily include natural language processing tools and methods [4]. Currently, the most popular areas in the field of text classification and analysis include machine learning (ML) methods and large language models. ML methods for text classification should be highlighted here [5,6]; among them, the use of TF-IDF (term frequency-inverse document frequency) analysis methods and neural network technologies prevail. Recently, the focus in the study of natural language processing has shifted to the field of large language models (LLMs) positioned as general methods for analyzing textual information and designed to solve a wide range of tasks. LLMs, such as BERT, the GPT family, and YaLM, have begun to actively replace techniques adapted to solving specific problems [7,8,9]. They are based on a neural network with a large number of parameters, trained on very large (huge) amounts of unlabeled data. LLMs are based on the architecture of transformers, and one of their most important features is multi-modal learning, which allows them to form more accurate representations based on data from different sources—text, audio, graphics, etc. [10]. Although such models are considered common for various text analysis tasks, they often require training. For example, BERT needs training to perform sentiment analysis. In addition, a major limitation of using LLMs is their high demand on computational resources.

Despite significant advances, they are usually not universal, and most text classification methods involve a number of limitations. To solve specific problems, LLMs and ML methods require the training of computational models and the processing of large amounts of data, and therefore significant computational resources. These limitations complicate their widespread practical application.

At present, the field of text data analysis, which is related to the use of methods of quantum probability theory, actively develops and promises good results to be obtained [11,12,13]. We especially highlight the impact of the project on quantum-like information retrieval (see [11,14]). In particular, a work [11] describes in detail the development of a quantum approach to text analysis and classification. In this field, quantum formalism is not considered a means of describing the subatomic world but a mathematical apparatus adapted to work under conditions of uncertainty. Therefore, the use of quantum mechanical methods can reduce the dimensionality of data or improve classification accuracy and successfully replace or complement ML methods and LLMs, reducing the resource intensity of text data analysis algorithms.

An alternative to ML in text classification tasks can be the use of heuristic (quantum-like or quantum-inspired) algorithms. The application of such algorithms is based on the use of interference effects for the wave function, which is well known in quantum mechanics. In particular, the quantum-like interference approach can be used to successfully explain certain effects in cognitive sciences and decision-making problems under uncertainty, e.g., [15,16,17,18]. The quantum-like interference approach becomes useful for big data analysis; here, we emphasize applications of quantum-like clustering for medical data used for diagnostics of neurological disorders, epilepsy, and schizophrenia [19].

The article is arranged as follows. Section 2 introduces the spherical wave-based model underlying our approach and describes the corresponding semantic text classification algorithm. Section 3 is divided into two subsections. Section 3.1 outlines the core of the experimental setup and compares the classification accuracy of the proposed method with that of conventional ML techniques. For this study, we used a dataset of 500 customer reviews from Amazon.com. Section 3.2 discusses the experimental results. The proposed method achieved relatively high classification performance, with an F-score of approximately 0.8. We also analyze the impact of interference effects in semantic space on classification quality. Section 4 addresses the issue of computational efficiency. By employing an integrated optimization strategy, we demonstrate how the algorithm’s time complexity can be reduced to O(n). In Section 5, we summarize the results that we obtained.

## 2. Theoretical Background

In this study, for text representation, we have developed the wave-like model, which is based on the concept of a wave function, $\psi (\vec{r})$; it allows text interpretation as a wave packet consisting of a group of waves (wave functions)—semantic units able to interfere with their semantics, cf. [20].

The model that we use below relates to spherical waves. In physics, they represent the solution of the Helmholtz equation (cf. [21,22,23])

(1) $$\left(\nabla^{2}+k^{2}\right)\psi \left(\vec{r}\right)=0,$$

where $\nabla^{2}$ is the Laplacian, k=ω/c is the wavenumber, and ω is the frequency. In general, the solution to (1) for the function $\psi (\vec{r})$ involves finding dependencies on three variables, r,θ, and φ, that relate to the spherical coordinates. In quantum mechanics, spherical waves are considered within scattering theory, cf. [21,24]. The variable *r*, for our purposes, specifies the semantic distance. However, the meaning of the phase variables in our problem is not so clear. We can exclude these variables from consideration if we assume that the function $\psi \left(\vec{r}\right)\equiv \psi (|\vec{r}\left|\right)$ depends on $r\equiv |\vec{r}|$; see e.g., [22,23]. In a more general case, we can consider solutions in the form of free spherical waves, which implies that the quantum orbital number is equal to zero.

Thus, the *spherical wave-like model* is specified in this work in the following form:

(2) $$\psi \left(r\right)=\frac{A}{r}e^{-ikr+i\phi_{0}},$$

where *A* is the amplitude, *r* is the distance from the source, and $\phi_{0}$ is the initial phase.

According to the quantum description [25], the wave function reflects the quantum-like state of the semantic unit as an object in a Hilbert space, and we can find an object at a given point in space with the following probability density:

(3) $$\rho \left(r\right)={\left|\psi \left(r\right)\right|}^{2}.$$

Notably, spherical wave-like model (2) enables accounting for the weakening of the probability density $\rho \left(r\right)\propto 1/r^{2}$. In a more general form, it is possible to assume that ρ(r) obeys a power-law distribution $\rho \left(r\right)\propto 1/r^{1+m}$, where m>0. Such probability density functions are capable of dealing with complex, highly fluctuating systems. These systems are encountered in our daily lives. They relate to the social sciences, economics, linguistics, etc.; see e.g., [26,27]. Notably, at a low *m*, the power-law distribution exhibits so-called “thick tails”, which contribute to essential differences in the behavior of complex systems compared to the familiar Gaussian distribution [28]. To be more specific, in this work, we examine the limit of m=1. In particular, the model described by (2) performed well with Russian-language data; cf. [29]. For this reason, we are using the same model for the English data. Notably, it is also possible to recognize *m* as an additional fitting parameter that enables the tuning of the probability density for the set of objects; see below. The solution to this problem is likely to be more complex, as it will involve adapting (optimizing) *m* to particular empirical data, cf. [30]. This issue will be examined in detail in future publications.

Considering the quantum states of a group of *M* waves, the probability density of detecting a set of objects at a given point in space is given as follows:

(4) $$\rho \left(r_{1},\ldots ,r_{M}\right)=\sum_{j=1}^{M}{\left(\frac{A_{j}}{r_{j}}\right)}^{2}+\Delta \left(r_{1},\ldots ,r_{M}\right),$$

where *M* is the number of objects, and we created a definition,

(5) $$\Delta \left(r_{1},\ldots ,r_{M}\right)=2\sum_{j=1}^{M-1}\sum_{n=j+1}^{M}\left(\frac{A_{j}A_{n}}{r_{j}r_{n}}\right)\cdot \cos\left(k_{j}r_{j}-k_{n}r_{n}\right)$$

In Equations (4) and (5), $\Delta \equiv \Delta (r_{1},\ldots ,r_{M})$ characterizes the contribution of quantum-like interference in total probability density; cf. [31]. In particular, interference of probabilities is constructive if Δ>0. The first sum in (4), that is

(6) $$\rho_{cl}\left(r_{1},\ldots ,r_{M}\right)=\sum_{j=1}^{M}{\left(\frac{A_{j}}{r_{j}}\right)}^{2},$$

gives the classical probability that we can recognize in the absence of interference. The possibility of employing a constructive interference of probabilities is one of the main distinguishing features of quantum probability calculus (see [32] in the discussion of the role of constructive interference in approaching supremacy in quantum calculations). Studying constructive interference of probabilities in semantic text classification can result in novel information technologies. We stress that our model needs no quantum computers; it is implemented on classical digital ones.

Equation (4), which characterizes the behavior of a wave packet in a Hilbert space, can be adapted to describe textual data in a semantic space by appropriate interpretation of parameters. In this case, the number of objects *M* is considered the number of non-repeating semantic units of the text, amplitude *A* is the number of repetitions of each semantic unit, distances $r_{j}$ specify the semantic distances from each semantic unit of the text to the point in space where the probability density, $\rho \equiv \rho (r_{1},\ldots ,r_{M})$, is calculated, wave numbers, $k_{j}$, are some parameters of the semantic units of the text, which we denote as “wave number” and which require separate consideration.

Wave numbers of semantic units cannot be determined directly from the text; however, they can be calculated on the basis of the following considerations. The function described by Equation (4) obviously involves an irregular set of maxima and minima, and the maxima correspond to the regions with the highest probability densities of detection of objects represented by this function.

Let us assume that we know a certain point at which the probability density should reach a maximum. For example, such a point can be the subject of the text if it is known in advance, as is often the case with sentiment analysis problems. Knowing this point, we can determine the distance, $r_{ci}$, to it from each word of the text, as the semantic distances between the corresponding concepts. If such a specific point cannot be found, the centroid of the text can be used as it. A text, as a set of words, can be represented in semantic space as a three-dimensional object; the relative distances between individual words determine the positions of the vertices. In this case, the distances from each word of the text to its centroid can be calculated using Formula (7):

(7) $$r_{ci}=\frac{\sum_{j=1}^{M}r_{ij}}{M},$$

where $r_{ij}$ is the semantic distance between two words of the text. The interference maximum is achieved when the phases of all wave functions at a given point are equal (8):

(8) $$k_{i}\cdot r_{ci}=const,$$

where const is some constant that represents a fitting parameter of the model. In this case, the wave numbers of each semantic unit can be calculated as follows:

(9) $$k_{i}=\frac{const}{r_{ci}},$$

where *k*_*i*_ is the wave number of the *i*-th word; *r*_*ci*_ is the distance from the *i*-th word to the maximum point of the function ρ.

In the calculations, we use const=1. This is based on the assumption that the text should be localized near a specific point chosen for calculating wave numbers. Furthermore, we assume that the main maximum of the ρ should be located at this point; the other maxima will then be in areas semantically close to the text.

**Figure 1:**
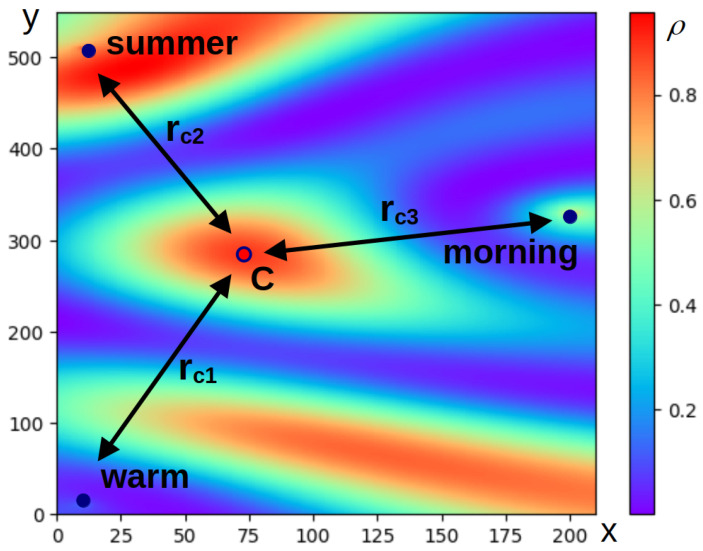
Color map of the probability density, ρ, calculated with the help of (4) for three wave functions corresponding to three words of the phrase. For visualization, the points reflecting the words of the phrase are placed on the plane; the axes of the plane relate to Cartesian coordinates (x,y) expressed in conventional units. The coordinates of the points have been selected in such a way that the distances between them are proportional to the semantic distances between the words. In particular, the distances between two points, $r_{ij}$, are specified as $\sqrt{{\left(x_{i}-x_{j}\right)}^{2}+{\left(y_{i}-y_{j}\right)}^{2}}$, where $\left(x_{i},y_{i}\right)$ and $\left(x_{j},y_{j}\right)$ are the coordinates of the corresponding points. The wave numbers are calculated using (7) and (9); for const=4, the $r_{ci}$ correspond to the semantic distance between the individual words of the phrase, and the centroid of the triangle reflects the relative position of the words in semantic space. The narrow color box reflects the probability density levels, represented in dimensionless units; purple corresponds to the minimum level of probability density, and red corresponds to its maximum.

To illustrate the described approach, the process of determining the wavenumbers in the phrase “warm summer morning” should be considered. Figure 1 shows the relative arrangement of the words of the phrase in question according to their mutual semantic distances, as well as a variant of the color map of function ρ. The coordinates along the axes of the plots and the levels of the color map are given in conventional units. The maximum point of the function ρ is the centroid of a triangle whose vertices are determined by the words of the phrase. The wave numbers of the words are calculated by Equations (7) and (9) for const=4. This value has been chosen to improve the visualization of the interference pattern that occurs when exploring an artificial phrase consisting of only three words. In this case, a less visible picture is obtained with lower values of const. The difference between *n* and the value used in the calculations made it possible to demonstrate the approach to calculating wave numbers clearly here.

To determine the semantic distances between the semantic units of a text, vector language models can be used, which allow for calculating the semantic proximity as the cosine distance between the corresponding terms. In this case, the semantic distance is the value inversely proportional to the semantic (cosine) proximity of the corresponding vectors. In this work, we also suppose that the wave function (2) is normalized; i.e.,

(10) $$\int_{V_{s}}{\left|\psi \left(r_{1},..,r_{M}\right)\right|}^{2}dV_{s}=1,$$

where $V_{s}$ is the volume of the semantic space containing all the meanings represented in a given language. In our case, the vector language model can be considered a relevant representation of the semantic space. So, Formula (10) can be represented as follows:

(11) $$\sum_{i=1}^{N_{vsm}}{\left|\psi \left(r_{1i},..,r_{Mi}\right)\right|}^{2}=1,$$

$N_{vsm}$ is the number of vectors in the vector language model. $r_{1i},\ldots ,r_{Mi}$ are the semantic distances between the vectors of the corresponding words of the text and the *i* vector of the language model.

Notably, in text classification tasks, including sentiment analysis, the basis for determining the most preferred classes is not the absolute value of probability density ρ but its ratio in the class domains, which automatically accounts for the normalization conditions (10) and (11).

Thus, we have described the wave model of the representation of textual information in the form of a set of waves. This model allows for estimating the probability density of text recognition in regions of semantic space defined by specified semantic concepts. This means that the model presented is suitable for classifying text in terms of semantic categories. One of these semantic categories can be the emotional tonality of the text, i.e., this model is suitable for solving problems of sentiment analysis.

**Figure 2:**
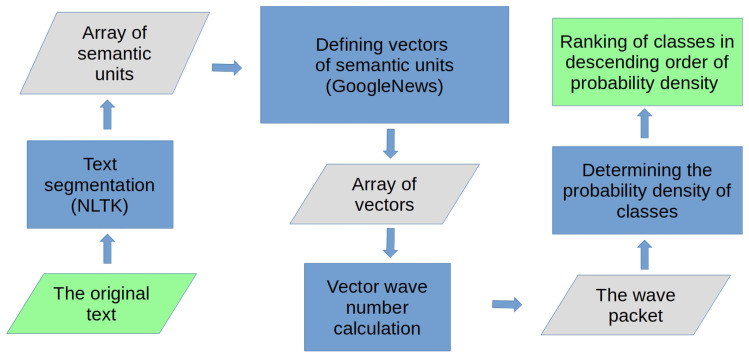
Block diagram of a semantic text classification algorithm. The input and output stages of the algorithm are marked in green, the data processing stages are blue, and the intermediate datasets are gray.

In this paper, we propose the following general workflow for text classification based on the proposed wave model of text; see Figure 2.

At the first step of the algorithm, the text is transformed into an array of semantic units. At this stage, it is important to take into account the peculiarities of the language of the text in question and its belonging to one of the types of language: synthetic, analytical, or agglutinative. The type of language determines the algorithm for segmenting the text into semantic units. For example, Russian belongs to the type of synthetic languages in which the main carriers of semantics are words, regardless of word order and environment in the text. For English, an analytical type of language, the order of words in a sentence, word combinations, phrases with structural words, and intonation are important.

Since, in Russian, the main carriers of semantics are individual words, simple lemmatization algorithms can be used to segment the text into semantic units, i.e., to split the text into individual words and convert them into normal form. For example, we can use the rulemma (url: https://github.com/Koziev/rulemma, accessed on 29 March 2025) or NLTK (Natural Language ToolKit) (url: https://www.nltk.org/, accessed on 29 March 2025) libraries for the Russian language.

For analytical languages, simple lemmatization can lead to a significant loss in the meaning of the text, embedded in the word order in the sentence and phrases, taking into account the structural words. Therefore, segmentation of the text into phrases, where punctuation marks, conjunctions, and other words separating semantic units of the text act as separators, seems more promising. In this case, the question arises of how to determine the vector of the semantic unit of the text, which is a phrase that arises when the vector language model used provides only vectors of individual words. The simplest, and as shown later, quite effective approach may be to represent the vector of a phrase as the sum of the vectors of the words included in it.

After determining the semantic units of the text and calculating the corresponding vectors, the maximum point of the probability density of text recognition can be set, and the wave numbers of the text can be calculated with (9). Then, the probability densities of text recognition in the areas of terms defining classes in the semantic space are calculated, and the classes are ranked in descending probability density. Thus, the task of semantic text classification is considered to be solved.

## 3. Results

### 3.1. Experiment

The task of sentiment analysis was chosen to test the algorithm. Previously, to solve this problem for Russian-language reviews, the authors of [29] showed the high accuracy of the wave model and its competitiveness in comparison with ML methods [33]. The achieved accuracy in determining the emotional tone of Russian language reviews and the comparison with other methods are shown in Table 1 and Table 2, respectively.

In this work, we examine the applicability of the quantum-like wave model of text representation to the sentiment analysis of English-language reviews. An experimental data set was collected from reviews on the website of the online store Amazon.com. The list of products and the links to the review pages are shown in Table 3. For each product, 50 positive and 50 critical reviews were selected. The data from the website was used to tag the dataset, where the reviews are grouped as “positive” or “critical”. In total, 500 product reviews were selected.

For the dataset described, a study was carried out on different algorithms to divide text into semantic units in terms of their impact on the accuracy of text classification using the wave representation model. As mentioned above, English belongs to the class of analytical languages, where the semantic units are not single words but phrases, taking into account the word order and intonation. This study aimed to determine the most appropriate algorithm for dividing text into semantic units for further classification using the wave model of text representation.

The following algorithms were considered in the work:

(1) The lemmatization of the text, as a result of which the text was divided into separate words reduced to the initial form, followed by the exclusion of service words. In this case, official words were considered insignificant, without their meaning, or neutral concerning both classes of emotional tonality assessment. In this case, individual words were considered as a semantic unit, similar to the algorithm previously used for the Russian language.
(2) The word sequences resulting from the processing described in item (1) were divided into trigrams, with the first word of the next triad being the last word of the previous triad. The resulting trigram arrays were then processed manually, eliminating clearly meaningless phrases, such as “like now after”, “off and many”, and the like.
(3) The original text was divided into phrases using punctuation, parentheses, dashes, quotation marks, conjunctions, the article “the”, and the adverb “so” as separators.
(4) Additional manual processing was carried out on phrase arrays obtained after the text described in item (3) was split. Long phrases were split into individual phrases.

The texts were transformed into arrays of semantic units. The vector language model GoogleNews-vectors-negative300 was used to compute the vectors of the semantic units of the text, url: https://drive.google.com/file/d/0B7XkCwpI5KDYNlNUTTlSS21pQmM, accessed on 20 March 2025). In the first case, the word vectors obtained as a result of lemmatization were used directly. In the second case, the trigram vector was calculated as the sum of the vectors of the words in the trigram. In the third and fourth cases, the phrases were lemmatized, and the vector of the phrase corresponded to the sum of the vectors of the words contained in the phrase.

The next stage of the work was to calculate the wave numbers of semantic units according to the methodology described above. The topic of reviews was used as a special point for the calculation of wave numbers: “*smartphone*”—for reviews about smartphones, and “clothes”—for reviews about clothes.

As a result, all the parameters necessary to perform the classification with the wave model of text representation were determined. The wave numbers of semantic text units were calculated, and the distances between semantic units and classes were calculated as values inversely proportional to the cosine proximity of the corresponding vectors.

**Figure 3:**
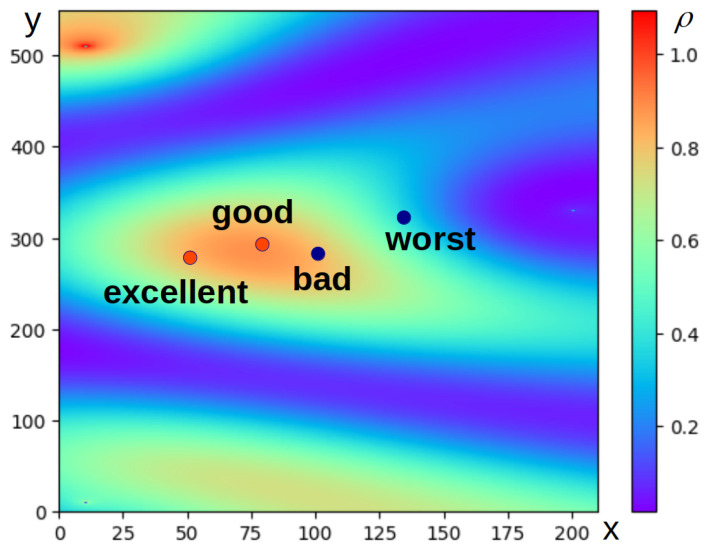
An example of a possible relative position of terms defining positive and negative classes (*excellent, good, bad, and worst*) on a color probability density map for a certain wave packet. It shows how, due to the high semantic proximity, similar probability density values can be obtained for the “*good*” and “*bad*” classes. Due to a greater mutual distance, “*Excellent*” and “*worst*” are more likely to fall into areas with different probability densities. For visualization, the points corresponding to the words are placed on the plane taking into account their mutual distances. The axes of the graph reflect Cartesian coordinates (x,y) expressed in conventional units.

The term “*excellent*” was chosen for the positive class and “*worst*” for the negative class. The terms corresponding to the classes in this problem were chosen based on two conditions: they should belong to a group of synonyms for a pair of terms, “*good*”–“*bad*”, and have a low semantic proximity at the same time. The second condition makes it possible to avoid the problem of the proximity of antonyms, which is typical for vector language models. In most cases, antonyms occur in the same contexts, which leads to the proximity of their vector representations. The pair of terms “*good*” and “*bad*” in the chosen vector language model has a proximity of 0.719, which makes them practically synonymous, unsuitable for designating polar classes. The pair “*excellent*” and “*poor*” has a cosine proximity of 0.157, and these terms can be used to denote positive and negative classes; this is illustrated in Figure 3.

Using the quantum-like wave model of text representation, the probability density of detecting the texts in the “*excellent*” and “*worst*” classes was calculated, and the class weights were defined with the following formula:

(12) $$p_{n}=\frac{\rho_{n}}{\sum_{i=1}^{C}\rho_{i}},$$

where *p*_*n*_ is the weight factor of a class with an index *n*, $\rho_{n}$ is the probability density of detecting the texts in the area of the class with the index *n*, and *C* is the number of classes.

The experimental results were established in Table 4.

Table 4 shows typical classification metrics, such as accuracy, precision, recall, and balanced F-score, which measure the quality of text classification, e.g., [34,35]. To calculate the metrics, the classification results were traditionally grouped by the following indicators: true positive (TP), true negative (TN), false positive (FP), and false negative (FN). The classification metrics were calculated using the formulas

(13) $$accuracy=\frac{TP+TN}{TP+TN+FP+FN},$$

(14) $$precision=\frac{TP}{TP+FP},$$

(15) $$reacall=\frac{TP}{TP+FN},$$

(16) $$F-score=2\cdot \frac{precision\cdot recall}{precision+recall},$$

Let us evaluate the influence of semantic interference on the classification result. In particular, to classify the texts, we examine only the term $\rho_{cl}$ in (4), assuming Δ=0, which we can recognize as a classical probability limit. Equation (6) reflects the Euclidean measure of semantic similarity between the semantic units of the text. Table 5 presents the results of the classification without taking into account the interference in semantics.

The set of experimental data and the classification results can be seen in the table on Google Drive (url: https://docs.google.com/spreadsheets/d/1TriUNo9DXS4Fu5ZiNlRG1VTUVb06TKsu, accessed on 29 March 2025). Here is a detailed description of the data placed in the “word collocation” columns of Table 4 and Table 5.

### 3.2. Discussion

As can be seen, the classification quality significantly depends on the algorithm used to segment text into semantic units. The worst results are obtained when text is split into individual words, while the best results are obtained when text is converted into an array of phrases with additional manual processing of the breakdown result. This confirms the aforementioned feature of analytical languages, such as English, namely, that semantic carriers are not individual words but phrases.

In the tasks of sentiment analysis, both positive and negative classes can be targeted for research. For example, in commerce, when evaluating product quality, the negative class is the target, and when evaluating the results of innovations, more attention is paid to the positive class. Therefore, Table 4 shows the metrics separately for both classes, as well as the average classification metrics. It can be seen that the quantum-like (wave) model, when splitting text into phrases, demonstrates fairly high accuracy (80.4%), precision, recall, and F-Score (about 0.8). At the same time, the results for the positive and negative classes are well balanced; the model gives no clear preference to any of the classes.

The bottom row in Table 4, reflecting the average F-score, is most indicative. We have used a balanced F-score here, which combines recall and precision with equal weights. The maximum value of the F-score is 1. Our result of 0.82 for a quantum-like text representation model in the case of splitting text into phrases can be considered a really strong indicator.

The results obtained allow us to consider the quantum-like (wave) model of text representation as a relevant classification method.

Table 5 contains the classification metrics similar to those shown in Table 4. The table demonstrates that, without the interference term, the accuracy reduces by 4–15%. Other classification metrics also decrease proportionally for both classes and all text segmentation options. Therefore, we can draw the conclusion that taking the interference into account noticeably affects the quality of text classification.

## 4. Optimization of Calculations

The classification of texts using the wave representation model and calculation of the probability density of the text proximity to the class according to Equation (4) leads to the complexity of the algorithm $O(n^{2})$, where *n* is the number of semantic units in the text. This complexity is explained by the presence of nested loops in the algorithm and the calculation of the cosine in the body of the loop.

Here, we are discussing possible optimization methods for the calculation of trigonometric functions in the body of nested loops and their potential impact on the time cost of the algorithm and the accuracy of the calculations.

To reduce the time required for the use of nested loops, computational parallelization was applied [36,37]. Since parallel computing in Python is associated with problems of exclusive access to resources through the GIL (Global Interpreter Lock) system, parallelization was performed through a multiprocessing system (not multi-threading) on a computer with a 10-core processor.

The use of parallel computing allows us to achieve significant results in data processing, not only in terms of speed and other indicators—for example, the scalability of the big data solution. Non-parallelized computations are usually performed in sequential mode on a single processor core, unless the compiler uses parallelization automatically.

Parallel computing provides an accuracy increase. This non-obvious result of parallelization is often overlooked because optimizing the computational process requires reducing the cost of data storage and processing; a possible solution in this case is to reduce the accuracy of data. For example, the number of decimal places can be decreased. Parallelization provides the processing of large values, and in some cases, this may be an opportunity to neglect storage savings.

Flexibility and the efficient use of resources are some of the current trends in computing. Due to parallelization, it is possible not only to use available resources efficiently but also to use cloud solutions freely.

To test the speed of data processing, we created a synthetic dataset containing 10 million parameter values of the wave model of text representation (wave numbers and proximity of semantic units). The data was stored in text format.

The data was processed in Python version 3.11, well known for its significantly optimized compiler for machine code.

We implemented the algorithm of the computation function of (4) in sequential and parallel. To generalize the solution used, the number of processes available for operation is read from the system data, and two processes are reserved for the needs of the operating system, so that the measurement indicators do not depend on the current system processes.

The calculations are distributed over the resulting number of processes. In this case, there were 12 processes available on a 6-core AMD Ryzen 5 5600H Radeon Graphics 3.30 GHz processor, of which 2 processes were excluded. It should be noted that parallelization between processes, rather than between cores, proved to be optimal, although it is clear that two processes on the same core already represent a parallelization option.

Several cosine optimization techniques were also investigated: the Taylor expansion $\cos\left(x\right)=\sum_{n=0}^{\infty }\frac{{\left(-1\right)}^{n}x^{2n}}{2n!}$ using the first two, four, and six terms (*n*), the use of pre-filled tables with values in 100, 1000, and 10,000 rows, and linear interpolation on the segments [0,π/2], [π/2,π]. The cosine calculation using the built-in function was used as a reference. For each of the optimization methods, the change in execution time (in % relative to the reference) and the maximum deviation of the calculated value from the reference value were estimated. The results are presented in Table 6.

The worst results were obtained for the Taylor expansion—high time cost with low accuracy. The use of the tabular method proved to be the most promising—it reduces the time cost by about 40%, and at the same time, the accuracy can be easily adjusted by changing the number of rows in Table 6. Linear interpolation provides a good performance boost, but it is characterized by a high error. A further reduction in time costs (about 10%) can be achieved by taking into account well-known properties of the cosine—equality to zero, one, and minus one at points that are multiples of π/2, even and odd numbers of π, respectively.

**Figure 4:**
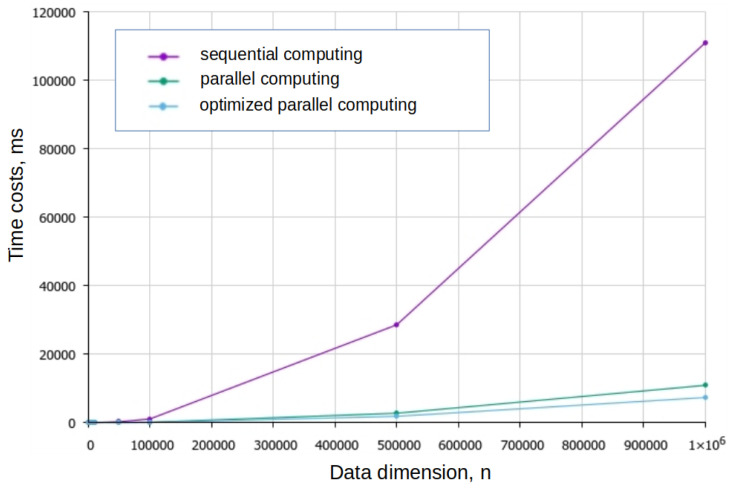
Dependence of the algorithm running time on the data dimension.

As a result, an integrated approach was used to optimize the algorithm, combining the use of parallel computing with a tabular method to calculate the cosine and take into account the known characteristics of the function. The plots of the dependence of the computation time on the dimension of the data are shown in Figure 4.

The use of parallel computing combined with optimization of cosine computation allows a significant reduction in time costs and achievement of a work intensity close to O(n). After optimization, the running time of the algorithm became proportional to $M^{0.88}$, where *M* is the number of words in the text.

## 5. Conclusions

We have studied the possibility of using a quantum-like (wave) model of text representation for the sentimental analysis of English-language customer reviews. The proposed model is based on the representation of semantic units of a text in the form of wave functions in a Hilbert space, and it allows for taking into account the interference of semantics. Based on this model, we have developed a text classification algorithm that uses a pre-trained universal vector language model requiring no additional training.

Our studies are based on previous experiments that demonstrated the effectiveness of the proposed model in sentiment analysis of Russian-language texts.

Five hundred customer reviews of the online store Amazon.com were randomly selected for our experiments. For the obtained data set, classification was carried out in the context of the classes “positive” and “negative” with the developed algorithm. According to the results of the experiments, the classification accuracy was 80%, and the precision, recall, and F-Score were about 0.8. At the same time, the classification metrics are well balanced for the negative and positive classes. A noticeable dependence of the classification quality on the algorithm for dividing the text into semantic units was revealed. The best result was obtained by splitting the text not into individual words, but into phrases, which confirms the features of the English language as belonging to the analytical class. However, to achieve the best possible classification, it was necessary to manually isolate semantically significant phrases. We have also studied the influence of semantic interference in a Hilbert space and on classification quality. To do this, the experimental dataset was classified without interference. As a result, it was found that the quantum-like interference effect leads to an increase of 15% in accuracy. Other classification metrics also increase proportionally. Thus, semantic interference has a significant impact on the quality of classification. A further improvement in the suggested algorithm requires an optimization procedure for interference phase Δ. We will consider this problem in further publications.

In this work, we took into account the peculiarities of the English language to classify the texts. In particular, it was demonstrated that classification metrics depend on the choice of algorithm used to divide text into semantic units. By examining various models of text decomposing into semantic units and using a quantum-like (wave) text representation for classification, we have achieved a classification accuracy of 80.4%. This indicator is even slightly better than the accuracy obtained earlier for the Russian language (79.3%), which successfully competes with the accuracy of ML methods.

We have also studied the reduction in time costs associated with algorithms based on the wave model of text representation. It was found that the proposed model lends itself well to optimization and that using an integrated approach—incorporating parallel computing and optimizing cosine calculation—reduces the estimated time costs from $O(n^{2})$ to O(n). This makes the algorithm more accessible for use in data analysis.

In general, the quantum-like (wave) text representation model has proven to be a worthy alternative to costly ML methods. This model needs no special training and uses a universal pre-trained vector language model; therefore, it does not require large machine and human resources for development and operation. At the same time, our model demonstrates high indicators of text classification quality metrics. Thus, we can conclude that the developed model can be used effectively in the tasks of text analysis and classification.

## Figures and Tables

**Table 1:** Accuracy of the quantum-like wave model of text representation for the task of sentimental analysis of Russian-language reviews, %.

Number of Classes	Accuracy
3 (positive, negative, neutral)	76.4
2 (positive, negative)	79.3

**Table 2:** Comparison of the accuracy of the wave model of text representation with the accuracy of ML methods for the task of sentiment analysis of Russian-language reviews, %.

Method	Accuracy
Decision tree	74.3
Logistic regression	76.2
Multi-layer perceptron	75.7
The wave model	79.3

**Table 3:** Data sources for collecting customer reviews.

Product
Moto G Play | 2024 | Unlocked | Made for US 4/64 GB | 50 MP Camera | Sapphire Blue
(url: https://www.amazon.com/Moto-Play-Unlocked-Camera-Sapphire/dp/B0CP6DDN1H, accessed on 29 March 2025)
Motorola Moto G Stylus | 2022 | 2-Day Battery | Unlocked | Made for US 4/128 GB |
50 MP Camera | Twilight Blue | 4G RAM, 4G/3G cellular technology
(url: https://www.amazon.com/Motorola-Stylus-Battery-Unlocked-Twilight/dp/B0CBNLS6BD, accessed on 29 March 2025)
Google Pixel 8—Unlocked Android Smartphone with Advanced Pixel Camera, 24-h Battery,
and Powerful Security—Hazel—256 GB
(url: https://www.amazon.com/Google-Pixel-Unlocked-Smartphone-Advanced/dp/B0CGT5CLJR, accessed on 29 March 2025)
Lee Women’s Ultra Lux Comfort with Flex Motion Bootcut Jean
(url: https://www.amazon.com/LEE-Womens-Regular-Bootcut-Renegade/dp/B07B6CVRNK, accessed on 29 March 2025)
Topdress Women’sVintage Polka Audrey Dress 1950s Halter Retro Cocktail Dress
(url: https://www.amazon.com/Topdress-Womens-Vintage-Cocktail-Black/dp/B078WLG8ZW, accessed on 29 March 2025)

**Table 4:** Text classification metrics using the wave representation model in a sentiment analysis problem, depending on the text segmentation method.

Semantic Unit	Word	Trigramm	Phrase	Word Collocation
accuracy	65.2	67.8	73.2	80.4
Metrics for “positive” class				
precision	0.69	0.68	0.76	0.75
recall	0.54	0.67	0.68	0.84
F-score	0.60	0.68	0.72	0.80
Metrics for “negative” class				
precision	0.62	0.68	0.71	0.86
recall	0.76	0.69	0.78	0.77
F-score	0.68	0.68	0.74	0.82
Average metrics				
precision	0.66	0.68	0.74	0.81
recall	0.65	0.68	0.73	0.81
F-score	0.64	0.68	0.74	0.82

**Table 5:** Metrics for text classification without semantics interference for the task of sentiment analysis.

Semantic Unit	Word	Trigramm	Phrase	Word Collocation
accuracy	60.4	64.2	65.2	65.4
Metrics for “positive” class				
precision	0.64	0.62	0.64	0.64
recall	0.47	0.71	0.70	0.70
F-score	0.54	0.66	0.67	0.67
Metrics for “negative” class				
precision	0.58	0.67	0.67	0.67
recall	0.74	0.57	0.61	0.61
F-score	0.65	0.62	0.64	0.64
Average metrics				
precision	0.61	0.65	0.66	0.66
recall	0.61	0.64	0.66	0.66
F-score	0.60	0.64	0.66	0.66

**Table 6:** Comparison of cosine calculation optimization methods.

Method	Time Costs (%)	Maximum Deviation
math.cos	100	0
Taylor 6	239	0.0001004702957823067
Taylor 4	106	0.023977787376392445
Taylor 2	63	1.1239099258720886
Table 100	58	0.035826794978996435
Table 1000	58	0.0042015594319668725
Table 10,000	58	0.0003627159973305094
Linear	43	0.21048888756342232

## Data Availability

All data used during this study are available within the article.
